# Analysis of E2E Delay and Wiring Harness in In-Vehicle Network with Zonal Architecture

**DOI:** 10.3390/s24103248

**Published:** 2024-05-20

**Authors:** Chulsun Park, Chengyu Cui, Sungkwon Park

**Affiliations:** Department of Electronic and Computer Engineering, Hanyang University, Seoul 04763, Republic of Korea; rottitti@hanyang.ac.kr (C.P.); cuichengyu@saicmotor.com (C.C.)

**Keywords:** in-vehicle network (IVN), zonal-based IVN architecture, domain-based IVN architecture, wiring harness, end-to-end delay

## Abstract

With recent advances in vehicle technologies, in-vehicle networks (IVNs) and wiring harnesses are becoming increasingly complex. To solve these challenges, the automotive industry has adopted a new zonal-based IVN architecture (ZIA) that connects electronic control units (ECUs) according to their physical locations. In this paper, we evaluate how the number of zones in the ZIA affects the end-to-end (E2E) delay and the characteristics of the wiring harnesses. We evaluate the impact of the number of zones on E2E delay through the OMNeT++ network simulator. In addition, we theoretically predict and analyze the impact of the number of zones on the wiring harnesses. Specifically, we use an asymptotic approach to analyze the total length and weight evolution of the wiring harnesses in ZIAs with 2, 4, 6, 8, and 10 zones by incrementally increasing the number of ECUs. We find that as the number of zones increases, the E2E delay increases, but the total length and weight of the wiring harnesses decreases. These results confirm that the ZIA effectively uses the wiring harnesses and mitigates network complexity within the vehicle.

## 1. Introduction

The recent advancements in vehicle technology aimed at enhancing driver safety and comfort, along with the introduction of vehicle-to-everything (V2X) and over-the-air (OTA) technologies, are rapidly increasing the bandwidth and computing demands in vehicles. Incorporating these technologies into vehicles necessitates advanced electronic control units (ECUs) containing advanced sensors and actuators. Today, luxury vehicles typically have more than 100 ECUs that manage core functions such as windows, doors, trunk, and lamps, as well as advanced driver assistance systems (ADAS), infotainment, and other systems [1,2]. However, as the number of ECUs installed in a vehicle rises, so does the total length and weight of the wiring harnesses. Wiring harnesses constitute one of the heaviest components in a vehicle, weighing tens of kilograms and spanning several kilometers in length [3]. Consequently, the proliferation of wiring harnesses has magnified the size and complexity of in-vehicle networks (IVNs) and the delay of data transfers. To solve the challenges posed by the escalating number of ECUs and enhance driver convenience and safety, a novel IVN architecture capable of integrating various technologies is imperative.

Over the past few decades, IVN architecture has continued to evolve with the introduction of various technologies into vehicles. Initially, a central-gateway-based IVN architecture was mainly used, but since 2020, it has been changing to a domain-based IVN architecture (DIA) [4,5]. The DIA has a structure in which ECUs with similar functions are grouped into domains. Because the DIA groups ECUs with similar functions, it provides the advantage of processing complex functions more quickly than previous IVN architectures [6]. However, because DIA does not take into account the physical locations of the ECU, it often requires long wiring harnesses between the ECU and each controller. As a result, as the number of ECUs increases, the total length and weight of the wiring harnesses also increase. Increased wiring harnesses can result in increased IVN complexity, longer data transfer times, increased vehicle weight, and increased costs.

To solve problems that may arise from the DIA, vehicle manufacturers are paying attention to the ZIA. The first characteristic of the ZIA is that it determines connections based on the physical locations of the ECUs rather than their functionality. In the ZIA, ECUs are grouped into several zones according to physical locations. As a result, the ZIA has the advantage of reducing the total length and weight of the wiring harnesses compared to the DIA, simplifying the IVN structure, and providing fast data transmission performance [7,8]. The second characteristic is its centralized structure. The high-performance computing unit (HPCU), located centrally in the ZIA, manages and controls the ECU. The device can handle applications requiring high computing power, such as autonomous driving and OTA.

One of the key factors of ZIA design is the number of zones. This is an important factor that can affect data transfer time, wiring harnesses, and architectural complexity. In previous research [9], we verified that the ZIA outperforms the DIA in data transmission and wiring harnesses aspects through simulations. However, since we designed the ZIA with six zones and conducted simulations, verification of how the number of zones affects it has yet to be performed. Therefore, in this paper, we design the ZIAs with 2, 4, 6, 8, and 10 zones and analyze the impact of each zone count on data transmission time and wiring harnesses. To analyze the impact on data transmission time, we design the ZIAs using the OMNeT++ network simulator and then measure the E2E delay of data generated by ECUs. Next, we abstract the ZIAs with 2, 4, 6, 8, and 10 zones to evaluate the impact on the wiring harnesses. Using the abstracted ZIAs, we evaluate the impact on the wiring harnesses as the number of zones changes. Additionally, we analyze the effects of increasing the number of ECUs from 20 to 120 using an asymptotic approach. Finally, we theoretically analyze and predict how efficiently the ZIA can reduce the wiring harnesses compared to the DIA.

This paper is organized as follows: Section 2 provides an overview of the evolution of IVN architectures. Section 3 describes the ZIA design methodology to analyze the impact of the number of zones on the E2E delay and the ZIA abstraction methodology to analyze the impact on the wiring harnesses. In Section 4, we present the results on the impact of changing zone numbers on E2E delay, wiring harnesses length, and weight. Finally, Section 5 discusses the conclusions drawn from the study and future research directions.

## 2. Related Research

### 2.1. Domain-Based In-Vehicle Network Architecture

**Figure 1:**
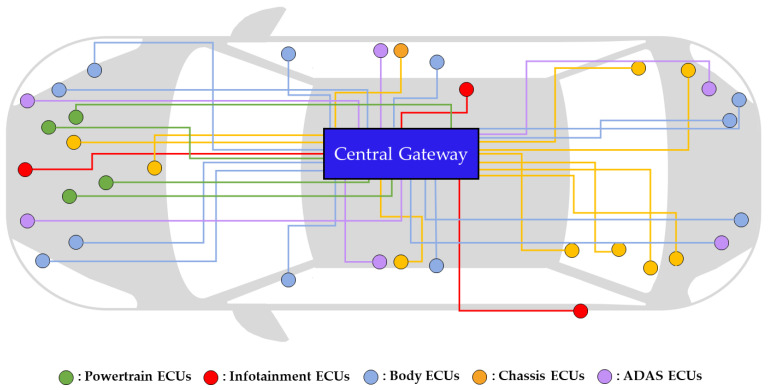
Example of central-gateway-based IVN architecture.

In the past, vehicles mainly used a central-gateway-based IVN architecture where all ECUs were connected to a central gateway. Figure 1 illustrates an example of a central-gateway-based IVN architecture. This architecture functioned effectively in vehicles with a limited number of ECUs and relatively small data volumes. However, the emergence of autonomous driving and OTA technologies has highlighted the limitations of this architecture as more diverse ECUs are added. Within this architecture, all data transmission and reception are routed through a central gateway, leading to a substantial load on the gateway. Consequently, problems such as data loss and delay may arise. Furthermore, the complexity of the IVN increases with the growing number of ECUs, as each ECU necessitates a connection to the central gateway. To solve these problems, the DIA was proposed.

**Figure 2:**
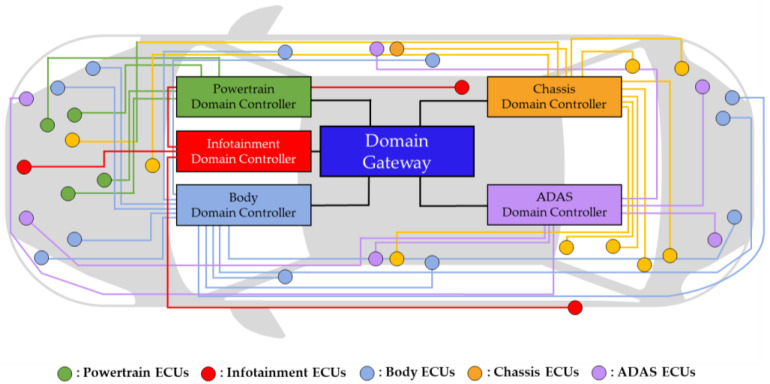
Example of domain-based IVN architecture grouped into five domains: PT, infotainment, body, chassis, and ADAS.

The DIA is widely adopted in modern vehicles and typically encompasses domains like powertrain (PT), infotainment, body, chassis, and ADAS. Figure 2 is an example of the DIA with a domain architecture applied to IVN. Due to its architectural design, the DIA can decrease the load on the central gateway compared to previous architectures. However, the DIA’s design does not account for the physical locations of ECUs when connecting them to the domain controller, resulting in increased wiring harnesses. While ECUs near the domain controller pose fewer challenges, those distributed around the vehicle, such as cameras, LiDAR, and radar, require extensive wiring connections. Consequently, the DIA may encounter problems related to wiring harnesses, architecture complexity, data transmission, vehicle weight, and cost. As various technologies are introduced into vehicles, a significant number of ECUs are expected to be added. To solve these problems in the DIA, a new, more advanced IVN architecture is needed.

### 2.2. Zonal-Based In-Vehicle Network Architecture

**Figure 3:**
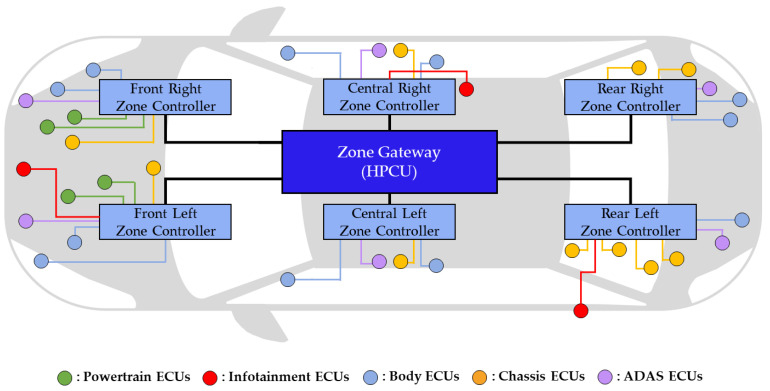
Example of zonal-based IVN architecture grouped into six zones.

To solve the problem of increasing the number of ECUs when applying the DIA in a vehicle, vehicle manufacturers have recently turned to the ZIA [10]. The ZIA groups ECUs into zones based on their physical locations, and Figure 3 illustrates an example of the ZIA grouping ECUs into six zones. This architecture groups physically adjacent ECUs into one zone, and the centralized HPCU efficiently manages all functions and devices in the vehicle. Each zone has multiple ECUs connected to one zone controller, and these controllers are connected to the HPCU. The ZIA has several advantages over the DIA. First, the length and weight of the wiring harnesses can be significantly reduced, simplifying the IVN structure compared to traditional architectures. Additionally, within each zone, the length of the wiring harnesses can be optimized by placing the zone controller in the center of each zone [11]. Second, by simplifying the IVN structure, the data transmission time can be shortened, which is especially important for time-sensitive applications such as autonomous driving. Finally, it can reduce wiring installation and maintenance costs, and the total cost of the vehicle [12]. In the ZIA design, the number of zones is believed to have a significant effect on IVN performance, vehicle weight, and cost. Accordingly, selecting the appropriate number of zones appears to be a key task in optimizing the IVN architecture and maximizing vehicle efficiency. According to [13], the number of zones when designing the ZIA is suggested to be 3 to 10.

## 3. Methodology for Performance Analysis

This section describes the methodology for analyzing the impact of the number of zones on data transfer time and wiring harnesses. First, to analyze the impact of the number of zones on data transfer time, we measure the E2E delay of time-sensitive IVN traffic. We design ZIAs consisting of 2, 4, 6, 8, and 10 zones to measure these delays using IVN traffic data from vehicle manufacturers and references [14,15].

Next, we abstract ZIAs with 2, 4, 6, 8, and 10 zones by referring to the actual vehicle dimensions. Using these ZIAs, we theoretically analyze the impact of the number of zones on the wiring harnesses. We also use an asymptotic approach to predict the impact on the total length and weight of the wiring harnesses as the number of ECUs increases from 20 to 120.

### 3.1. Design of IVN Architecture for E2E Delay

**Figure 4:**
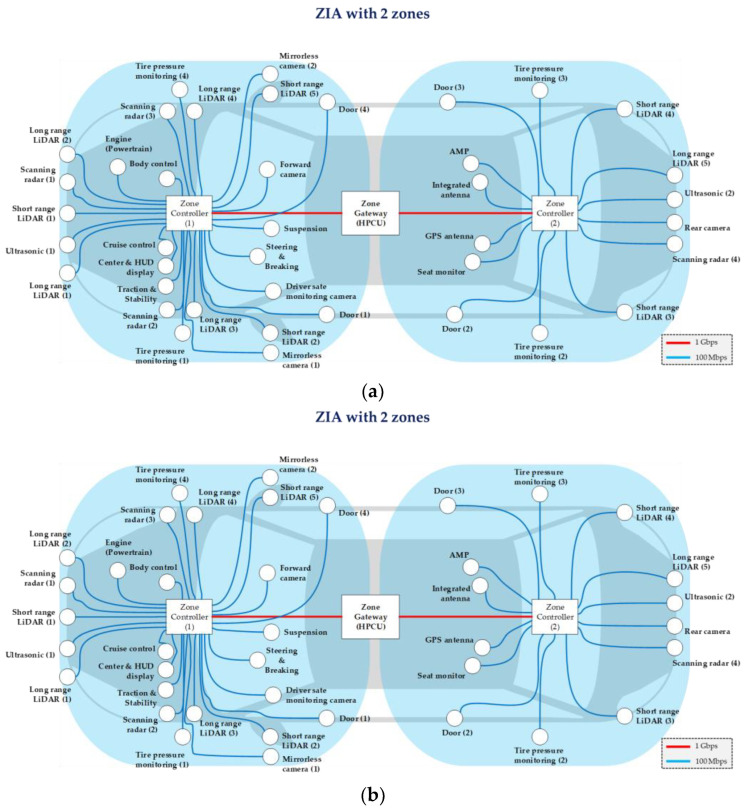
ZIAS: (**a**) ZIA with 2 zones and (**b**) ZIA with 4 zones.

**Figure 5:**
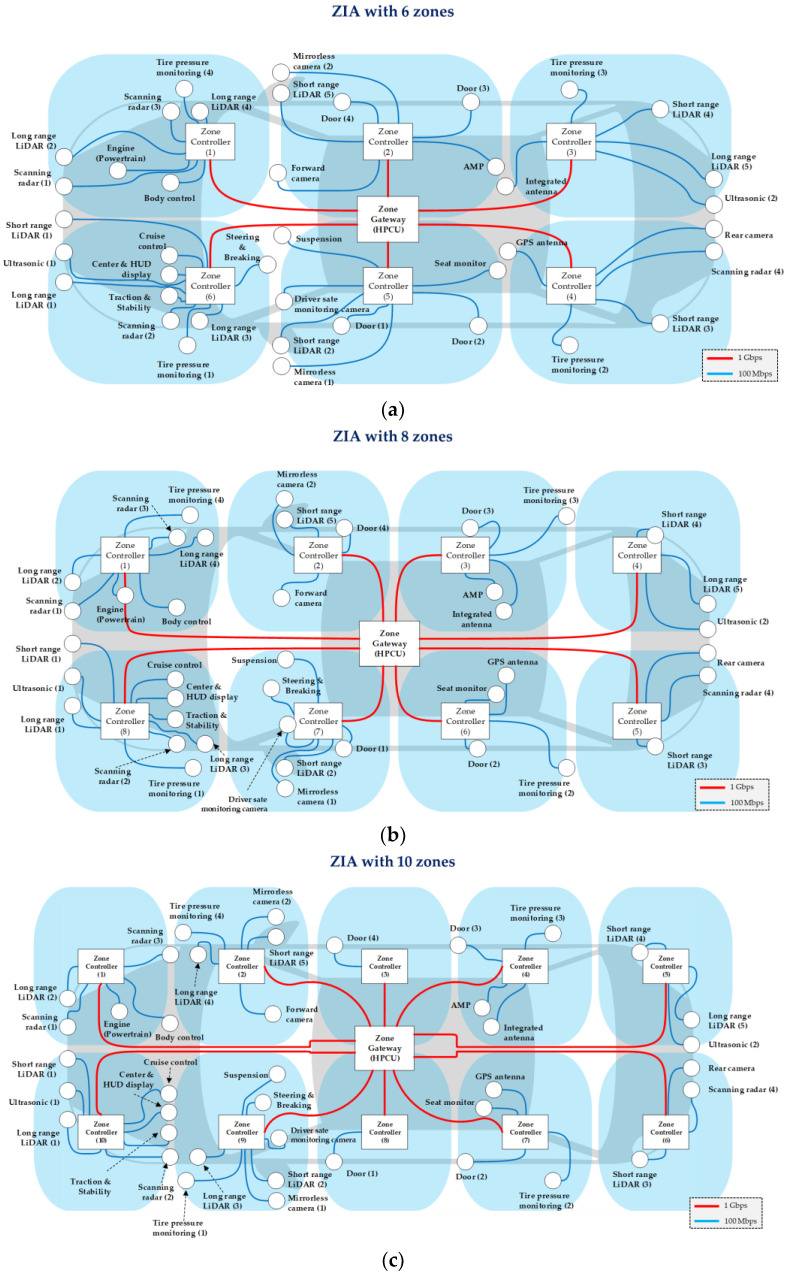
ZIAS: (**a**) ZIA with 6 zones, (**b**) ZIA with 8 zones, and (**c**) ZIA with 10 zones.

We design the ZIAs with multiple zones to measure the E2E delay of time-sensitive IVN traffic. Figure 4 shows the ZIAs with 2 and 4 zones, and Figure 5 shows the ZIAs with 6, 8, and 10 zones. The ZIAs designed in Figure 4 and Figure 5 are based on an automotive Ethernet protocol. In the past, vehicles have used local interconnect networks (LIN), controller area networks (CAN), and media-oriented system transport (MOST). However, with the introduction of autonomous driving, infotainment, and OTA technologies, IVNs have high communication requirements, including high data rates and low delay. Since the existing vehicle communication protocol technologies cannot meet these requirements, automotive Ethernet technologies, such as the IEEE 802.1 time-sensitive networking (TSN) standards, were developed [16]. We design and simulate a ZIA based on the TSN standard. When designing the ZIA, we did not use all the ECUs found in actual vehicles, which range from ECUs with simple functions such as windows, doors, seats, and trunk to ECUs with advanced functions related to autonomous driving such as sensors, cameras, LiDAR, and radar. In this paper, we design the ZIAs shown in Figure 4 and Figure 5 using 42 ECUs related to autonomous driving and infotainment, referring to the ECU locations mounted in actual vehicles [17,18,19].

### 3.2. Abstraction of IVN Architectures for Wiring Harnesses

**Figure 6:**
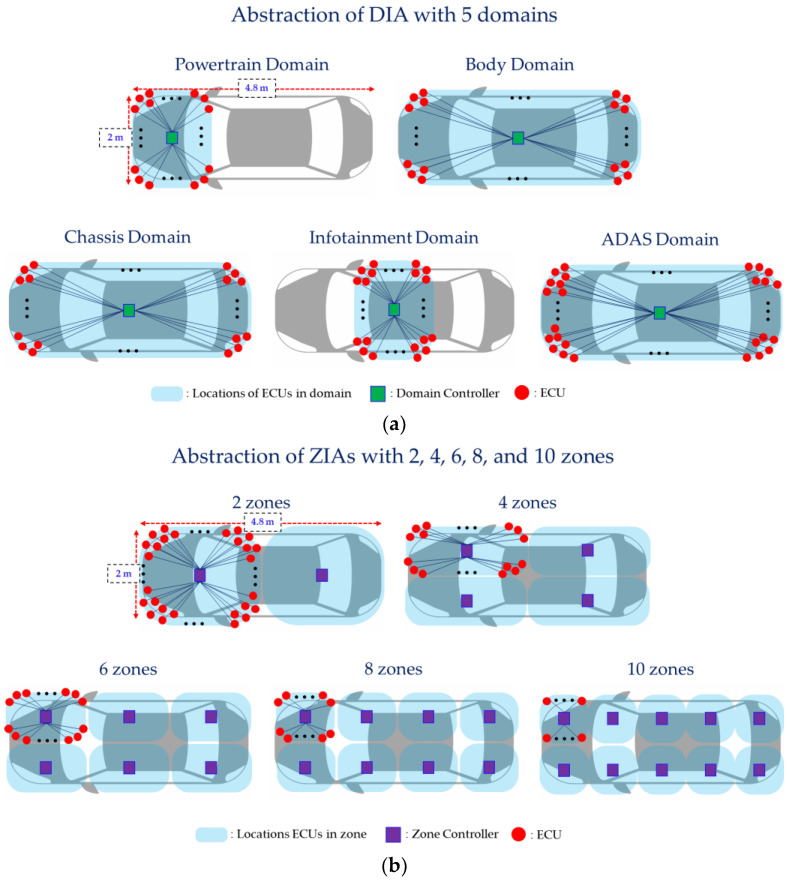
DIA and ZIAs abstracted to analyze the effect on the wiring harnesses according to changes in the number of zones: (**a**) DIA with 5 domains and (**b**) ZIAs with 2, 4, 6, 8, and 10 zones.

We predict and analyze the impact on the wiring harnesses when incrementally increasing the number of ECUs from 20 to 120 in the ZIAs with 2, 4, 6, 8, and 10 zones. Also, we analyze how the ZIAs with 2, 4, 6, 8, and 10 zones improve the wiring harness compared to the DIA with 5 domains. We abstract the DIA and ZIAs, as shown in Figure 6, based on a medium-sized vehicle that is 4.8 m long and 2 m wide. During the abstraction process, we make several assumptions. We know that each actual vehicle has its own domain and zone sizes, ECU and controller locations, and wiring harness shapes in the vehicle. We make the following assumptions to simplify the abstracted IVN architecture and ease computation.

In actual vehicles, the complexity and variability of wiring harnesses comprising numerous parts with diverse lengths, weights, and configurations pose challenges for direct comparison. For this analysis, we simplify by assuming uniformity in the number of parts and a linear connection between them, focusing on the zone impact.

Second, we consider the size and location of the zones and the distribution of ECUs within the zones. In the abstracted ZIAs, the zones are assumed to be the same size. For example, all zones are the same size in the ZIA with 4 zones. Then, within each zone, we calculate the total length and weight of the wiring harnesses, assuming the controller is centered, and the ECUs are located at the maximum distance from the controller. Finally, we assume that the ECUs in the ZIA are primarily located in the front of the vehicle. For example, in a ZIA with two zones and 20 ECUs, we assume that 12 ECUs are in the front of the vehicle and 8 ECUs are in the rear, reflecting the fact that critical components such as CAM, LiDAR, and radar are concentrated in the front of the vehicle.

Finally, the size and location of each domain in the DIA, and the number and locations of ECUs in each domain are approached. We referenced the locations of ECUs in the 5 domains of an actual vehicle. PT-related ECUs are mainly located in the front of the vehicle; infotainment-related ECUs are mainly located in the center of the vehicle; and body, chassis, and ADAS-related ECUs are evenly distributed throughout the vehicle. In particular, ADAS-related ECUs are assumed to have a higher number of ECUs compared to other domains. Also, within each domain, the length and weight are calculated, assuming that the domain controller is located in the center and the ECUs are located at the farthest distance.

## 4. Results for Simulation and Performance Analysis

This section describes the results of measuring the E2E delay and the total length and weight of the wiring harnesses using the designed ZIAs and abstracted DIA and ZIAs.

### 4.1. End-to-End Delay

#### 4.1.1. Simulation Environment

**Figure 7:**
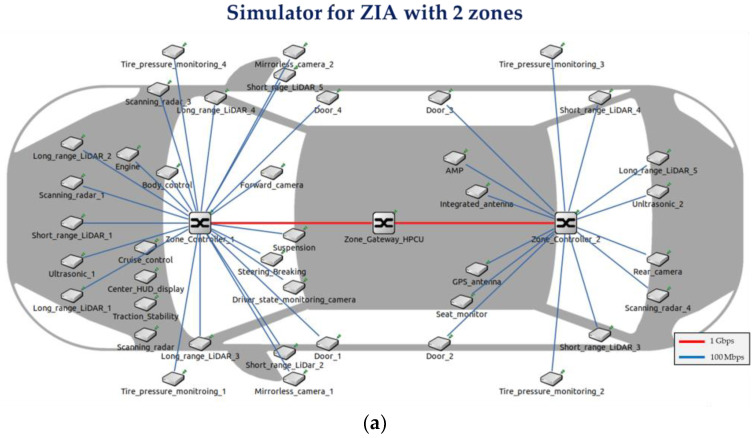

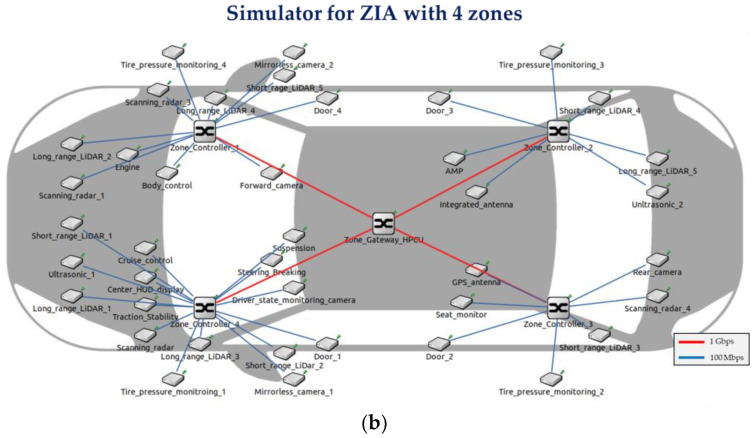
Simulators for the ZIAs developed with the OMNeT++: (**a**) ZIA simulator with two zones and (**b**) ZIA simulator with four zones.

**Figure 8:**
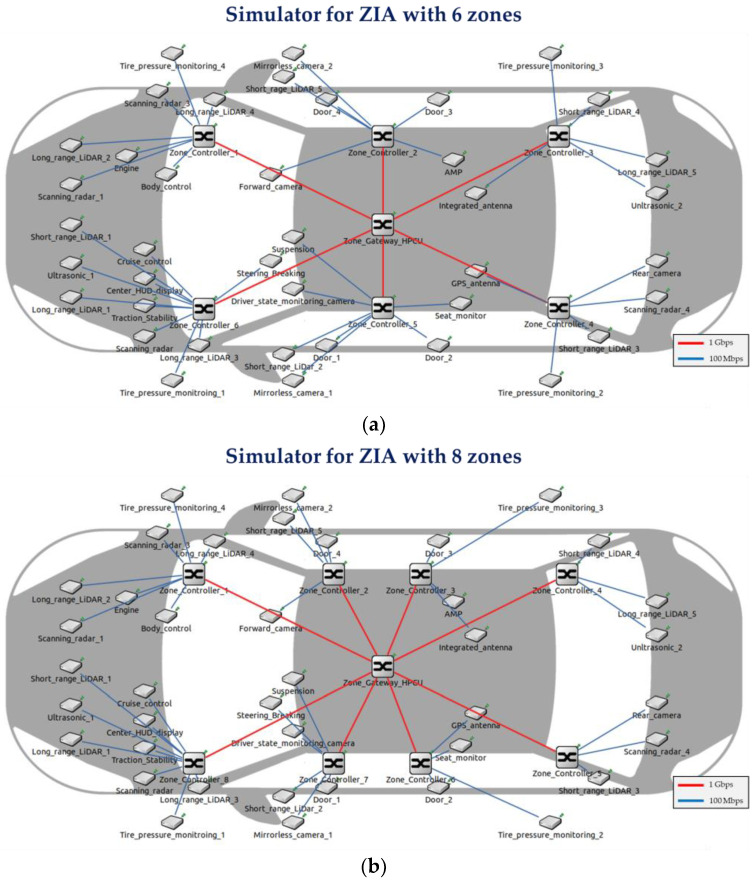

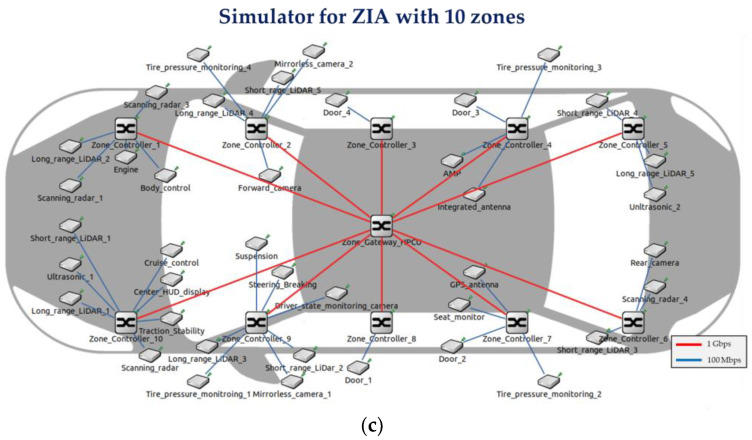
Simulators for the ZIAs developed with the OMNeT++: (**a**) ZIA simulator with 6 zones, (**b**) ZIA simulator with 8 zones, and (**c**) ZIA simulator with 10 zones.

We develop simulators to measure the E2E delay of time-sensitive IVN traffic utilizing the OMNeT++ version 5.6.7 [20] and the CoRE4INET framework [21]. The simulators are based on CoRE4INET and comply with the IEEE 802.1 TSN standards. The simulators developed for the previously designed ZIAs are for ZIAs with 2, 4, 6, 8, and 10 zones. Figure 7 shows the simulators for ZIAs with 2 and 4 zones, while Figure 8 shows the simulators for ZIAs with 6, 8, and 10 zones.

#### 4.1.2. Simulation Method

We refer to the traffic information provided by the vehicle manufacturers and [22,23,24,25] define the IVN traffic generated by the ECUs. Based on this, we measure the E2E delay for a total of 10 different IVN traffic in our simulation. Table 1 shows the IVN traffic flows and requirements between ECUs for this purpose. The IVN traffic is divided into four classes according to priority, each of which is categorized as ST, SR classes A and B, and BE according to the IEEE 802.1 TSN standards. High-priority data, such as command and control and LiDAR data, are assigned to the ST class, while medium-priority data, such as video streams and V2X data, are assigned to SR Class A. The lowest priority data, entertainment and upload data, are assigned to BE. The zone controller prioritizes transmitting high-priority data and uses the time-aware shaper (TAS) technology [26] defined in the IEEE 802.1 Qbv standard.

The total E2E delay for IVN traffic can expressed as in Equation (1) [27].

(1) $$d_{total}=d_{queue}+d_{trans}+d_{proc}+d_{prop}$$

Here, the total E2E delay $d_{total}$ can be divided into four parts as follows: queuing delay ($d_{queue}$), transmission delay ($d_{trans}$), processing delay ($d_{proc}$), and propagation delay ($d_{prop}$). Because the propagation speed of electromagnetic waves is similar to the speed of light, $d_{prop}$ is usually ignored in delay measurements. In the E2E delay measurement simulation, 100 pieces of data per IVN traffic are generated and transmitted; then, the E2E delay is measured, and the average value is calculated.

#### 4.1.3. Results

**Figure 9:**
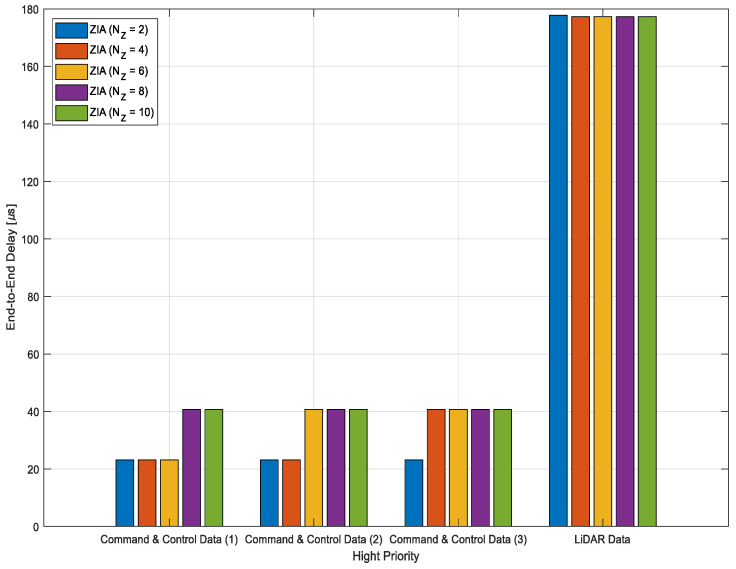
Average E2E delay for highest priority IVN traffic.

**Figure 10:**
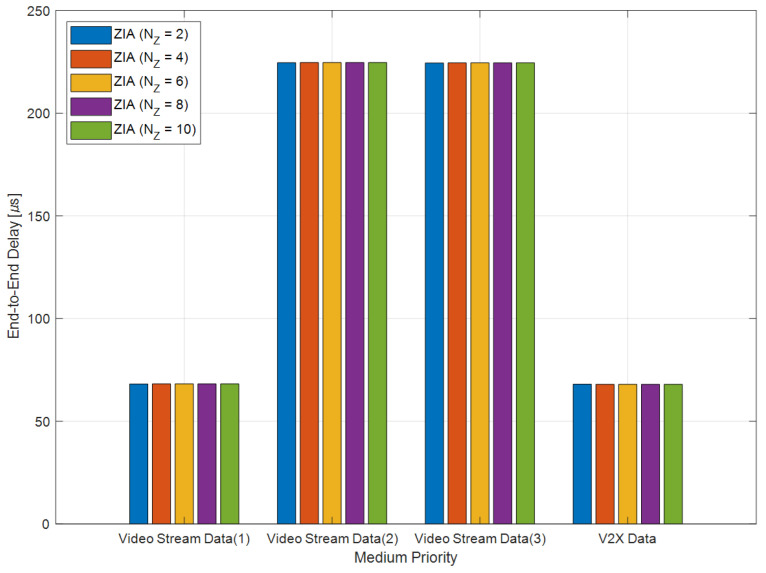
Average E2E delay for medium priority IVN traffic.

**Figure 11:**
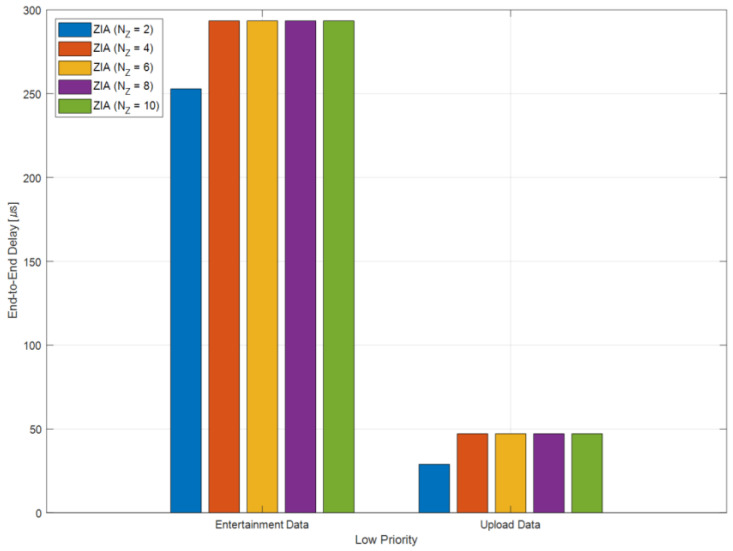
Average E2E delay for low-priority IVN traffic.

Figure 9, Figure 10 and Figure 11 show the average E2E delay results for 10 different IVN traffic. Figure 9 shows the average E2E delay results for command-and-control and LiDAR data, which have the highest priority. For the command-and-control data (1), the average E2E delay was 23.14 μs for ZIAs with two, four, and six zones, respectively. However, for ZIAs with 8 and 10 zones, the average E2E delay increased by about 76% to 40.74 μs. For the command-and-control data (2) and (3), the average E2E delay tended to increase as the number of zones increased. On the other hand, the LiDAR data showed similar average E2E delays regardless of the number of zones. Due to the larger traffic size, LiDAR data had a relatively higher average E2E delay than command and control data.

Simulations have shown that the number of zones affects the transmission of high-priority traffic. As the number of zones increases, the E2E delay for command-and-control data (1), (2), and (3) tends to increase due to the larger number of zone controllers that the traffic traverses. Furthermore, the E2E delay remains consistent regardless of the number of zones because the traffic passes through the same number of zone controllers. The command-and-control data are affected by an increase in the number of zones, while LiDAR data are not affected by an increase in the number of zones.

Figure 10 shows the average E2E delay results for video stream and V2X data with medium priority. For video stream (1), the average E2E delay was 68.18 ms in the ZIA with two zones. However, it increases slightly to 68.22 ms in the ZIAs with 4, 6, 8, and 10 zones. For the video stream (2), ZIAs with 2, 4, 6, 8, and 10 zones showed similar average E2E delays. For the video stream (3), the average E2E delay was measured to be 224.45 ms for ZIAs with two zones, but it increased to 224.49 ms for ZIAs with 4, 6, 8, and 10 zones, showing an increase of 0.04 ms. Finally, for the V2X data, similar average E2E delays were measured for ZIAs with 2, 4, 6, 8, and 10 zones. These simulated E2E delay results indicate that video stream (1) and (3) are affected by an increase in the number of zones, while video stream (2) and V2X data are not affected by an increase in the number of zones.

Figure 11 shows the average E2E delay of low-priority IVN traffic, which includes entertainment and upload data. For entertainment data, the average E2E delay was measured to be 252.9 μs for ZIAs with two zones, increasing to approximately 293.48 μs for ZIAs with 4, 6, 8, and 10 zones, representing a 16% increase. Regarding upload data, the average E2E delay was 28.9 μs for ZIAs with two zones, while for ZIAs with 4, 6, 8, and 10 zones, it increased to approximately 47.08 μs, showing a rise of about 62%. These results show that the E2E delays for entertainment and upload data increase with more than four zones. When transmitting low priority traffic, ZIA with two zones is the most efficient.

### 4.2. Total Length and Weight of Wiring Harnesses

#### 4.2.1. Calculation method with Asymptotic Approach

We utilize the abstracted DIA and ZIAs from earlier to analyze how the number of zones affects the wiring harnesses while also examining how the total length and weight of the wiring harnesses change as the number of ECUs increases from 20 to 120. Ultimately, we numerically compare and analyze how much more efficient the ZIA is compared to the DIA in terms of wiring harness aspects. The formula to calculate the total length of the wiring harnesses ($L_{W}^{DIA}$) in the DIA can be expressed as in Equation (2).

(2) $$L_{W}^{DIA}=\sum_{i=1}^{N_{D}^{i}}l_{D}(i)+\sum_{i=1}^{N_{D}^{i}}\sum_{j=1}^{N_{E}^{j}}l_{E}(i,\;j)$$

Here, $L_{W}^{DIA}$ is divided into two parts. The first part $\sum_{i=1}^{N_{D}^{i}}l_{D}(i)$ represents the total of the wiring harnesses between the central gateway and the domain controllers. The second part $\sum_{i=1}^{N_{D}^{i}}\sum_{j=1}^{N_{E}^{j}}l_{E}(i,\;j)$ represents the total length of the wiring harnesses between the domain controllers and the ECUs.

In $\sum_{i=1}^{N_{D}^{i}}l_{D}(i)$, $N_{D}^{i}$ represents the number of domain controllers, and $l_{D}(i)$ is the length between the central gateway and $i^{th}$ domain controller. In $\sum_{i=1}^{N_{D}^{i}}\sum_{j=1}^{N_{E}^{j}}l_{E}(i,\;j)$, $N_{E}^{j}$ represents the number of ECUs, and $l_{E}(i,\;j)$ is the length between the $i^{th}$ domain controller and the $j^{th}$ ECU.

The total length of the wiring harnesses in the ZIA can be expressed as in Equation (3).

(3) $$L_{W}^{ZIA}=\sum_{i=1}^{N_{Z}^{i}}l_{Z}(i)+\sum_{i=1}^{N_{Z}^{i}}\sum_{j=1}^{N_{E}^{j}}l_{E}(i,\;j)$$

Here, the total length of the wiring harnesses in the ZIA ($L_{W}^{ZIA}$) is divided into two parts. The first part $\sum_{i=1}^{N_{Z}^{i}}l_{Z}(i)$ is the total length of the wiring harnesses between the central gateway and the zone controller. The second part $\sum_{i=1}^{N_{Z}^{i}}\sum_{j=1}^{N_{E}^{j}}l_{E}(i,\;j)$ is the total length of the wiring harness between the zone controller and the ECU.

In $\sum_{i=1}^{N_{Z}^{i}}l_{Z}(i)$, $N_{Z}^{i}$ is the number of zone controllers, and $l_{Z}(i)$ is the length between the central gateway and the $i^{th}$ zone controllers. In $\sum_{i=1}^{N_{Z}^{i}}\sum_{j=1}^{N_{E}^{j}}l_{E}(i,\;j)$, $N_{E}^{j}$ is the number of ECUs, and $l_{E}(i,\;j)$ is the length between the $i^{th}$ zone controller and the $j^{th}$ ECU.

In Equations (2) and (3), we assume the length between the central gateway and each controller is about one-third of the length of the vehicle (about 1.6 m). This assumption was made because it is difficult to measure it in practice, and because the actual distance between the central gateway and the controllers can vary significantly depending on the design and construction of the vehicle. Therefore, this assumption was made to simplify the model and facilitate calculations.

The total length and weight of the wiring harnesses in the DIA and ZIA can be expressed as in Equations (4) and (5), respectively.

(4) $$W_{W}^{DIA}=L_{W}^{DIA}\times w_{f}$$

(5) $$W_{W}^{ZIA}=L_{W}^{ZIA}\times w_{f}$$

Here, $W_{W}^{DIA}$ and $W_{W}^{ZIA}$ can be calculated by multiplying Equations (2) and (3) by the wiring harness weight per meter ($w_{f}$), respectively. According to [28], the total length and weight of the wiring harnesses used in vehicles are up to 1.6 km and 60 kg, respectively. In this paper, we assume $w_{f}$ to be 0.0375 kg and calculate $W_{W}^{DIA}$ and $W_{W}^{ZIA}$. Table 2 shows the parameters we organized to calculate the total length and weight of the wiring harnesses from the DIA and ZIA using the asymptotic approach.

#### 4.2.2. Results

**Figure 12:**
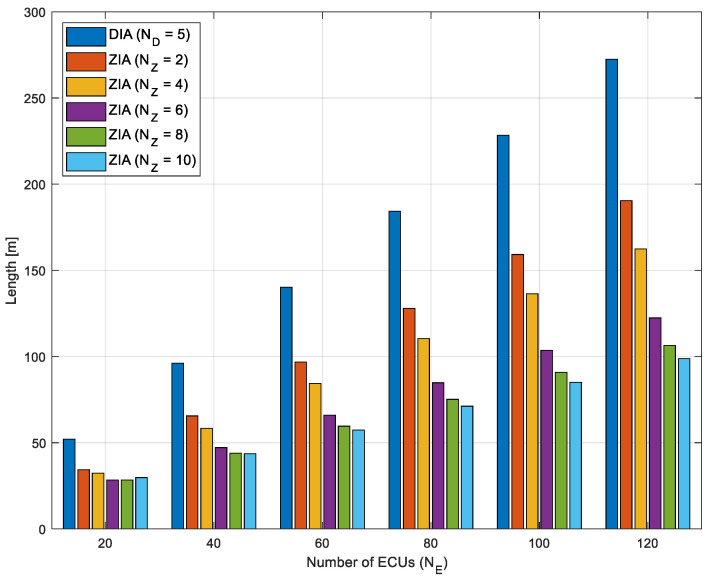
Total length of the wiring harnesses as the number of ECUs increases from 20 to 120 in ZIAs with 2, 4, 6, 8, and 10 zones, and the DIA with five domains.

Figure 12 shows the total length of the wiring harnesses as the number of ECUs increases from 20 to 120 in the ZIAs with 2, 4, 6, 8, and 10 zones, and the DIA with five domains. The results show that the ZIA with three zones can reduce the total length of the wiring harnesses by 33.9%, 31.8%, 30.9%, 30.6%, 30.3%, and 30.1% when the number of ECUs is 20, 40, 60, 80, 100, and 120, respectively, compared to the DIA. The ZIA with four zones reduces the total length of the wiring harness by 33.9%, 31.8%, 30.9%, 30.6%, 30.3%, and 30.1% compared to DIA for ECU counts of 20, 40, 60, 80, 100, and 120, respectively. The ZIA with six zones has 45.5%, 50.9%, 52.9%, 53.9%, 54.6%, and 55.1% less total length than the DIA. The ZIA with eight zones reduces the total length by 45.5%, 54.2%, 57.5%, 59.2%, 60.3%, and 61% compared to the DIA. Finally, the ZIA with 10 zones exhibits a reduction of 42.7%, 43.6%, 57.4%, 62.8%, and 63.7% compared to DIA for ECU counts of 20, 40, 60, 80, 100, and 120, respectively. The percentage reduction in wiring harness length tended to be higher as the number of zones increased, which can be interpreted as more ECUs being able to be connected using shorter wiring harnesses.

Interestingly, the reduction rate in the ZIA with 10 zones when the ECU count is at 20 is lower than that in the ZIA with 8 zones. This suggests that as the number of zone controllers increases, the lengths of the wiring harnesses connecting the HPCU to the zone controllers also increase, resulting in a decreased reduction rate despite the higher number of zones. Therefore, deploying a ZIA with 10 zones appears to be more beneficial when the number of ECUs exceeds 40, optimizing the balance between zone quantity and harness length reduction.

**Figure 13:**
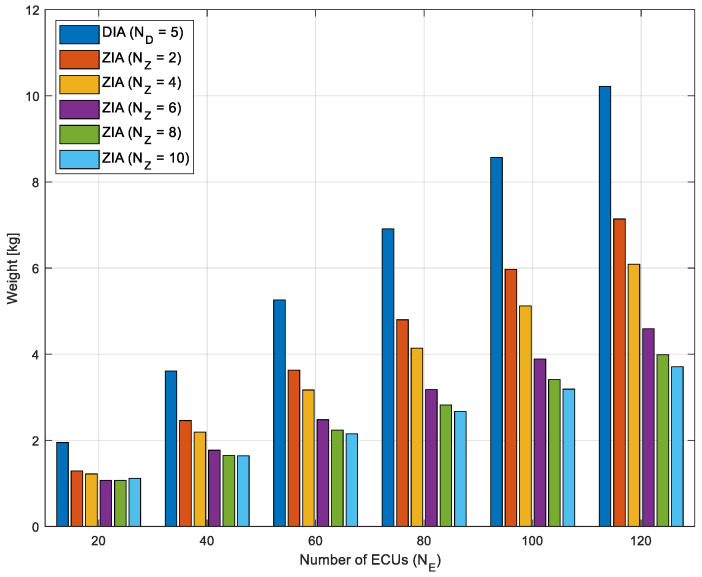
Total weight of the wiring harnesses as the number of ECUs increases from 20 to 120 in ZIAs with 2, 4, 6, 8, and 10 zones, and the DIA with five domains.

Figure 13 illustrates the anticipated weight of the wiring harnesses as the number of ECUs increases from 20 to 120 in the ZIAs with 2, 4, 6, 8, and 10 zones, and in the DIA with five domains. Overall, it is verified that the ZIAs can more efficiently reduce the weight of the wiring harnesses compared to the DIA. Particularly, there is a trend of increasing weight reduction efficiency as the number of zones increases.

## 5. Conclusions and Future Works

To provide safety and convenience for drivers, vehicles are being equipped with more ECUs than ever before. This increases the complexity, vehicle weight, and cost of IVNs and causes data transfer delays. Vehicle manufacturers are beginning to turn to the ZIA to solve these challenges.

This paper contributes to the understanding and advancement of ZIAs through the following:

- E2E delay analysis: this analysis measures the impact of the number of zones within the ZIA on E2E delay to help design ZIAs that efficiently transmit high-priority data.
- Wiring harness analysis: this analysis quantifies how the ZIA can effectively reduce the length and weight of the wiring harness, contributing to the design of more efficient ZIA configurations.
- Comparison analysis with DIA: contributes to the transition from the DIA to ZIA by providing results on the efficiency benefits of the ZIA over traditional DIA.
- Scalability analysis: contributes to the design of scalable vehicle network architectures by analyzing the impact of increasing ECU count on network performance.

In this paper, we analyzed the impact of applying a zonal architecture to an IVN on E2E delay and wiring harnesses. In particular, we focused on the impact of the number of zones in the ZIA design. We developed the simulators for ZIAs using the OMNeT++ and measured the E2E delay of the IVN to analyze the impact of the number of zones on the data transfer time. In addition, we abstracted ZIAs with 2, 4, 6, 8, and 10 zones to analyze the impact on the wiring harnesses. This allowed us to predict and analyze the change in the total length and weight of the wiring harnesses as the number of ECUs increased.

Using the simulators for ZIAs, we verified that the E2E delay increases as the number of zones increases. As the number of zones increases, the number of zone controllers that the IVN traffic has to pass through when it is transmitted also increases; so, the E2E delay tends to increase. On the other hand, we verified that the total length and weight of the wiring harnesses decrease as the number of zones increases. Therefore, vehicle manufacturers should choose the right number of zones to manage the vehicle’s internal network efficiently by optimizing the total length and weight of the wiring harnesses while minimizing the E2E delay.

Through this paper, we verified that applying the ZIA to vehicles is essential for efficiently organizing IVN architectures. However, a sudden change from the DIA to the ZIA requires a complete change in the vehicle development system. As a result, vehicle manufacturers may encounter cost and technical challenges. Therefore, as a future research direction, we believe it is necessary to study hybrid IVN architecture (HIA), which is an intermediate step between the DIA and ZIA, before applying the ZIA to vehicles. In the future, we will design the HIA and conduct a performance comparison study between the DIA and the ZIA regarding data transmission and wiring harnesses.

## Figures and Tables

**Table 1:** IVN traffic flows and corresponding requirements for the E2E delay measurement.

IVN Traffic Types	Priority	Payload Size [Byte]	Traffic Interval [μs]	Transmission Path
Command and control data (1)	High (ST)	64	500	Cruise control → Steering and breaking
Command and control data (2)	High (ST)	64	500	Cruise control → Suspension
Command and control data (3)	High (ST)	64	500	Cruise control → Engine
LiDAR data	High (ST)	512	500	Long range LiDAR (5) → Cruise control
Video stream (1)	Medium (SR class A)	1500	125	Forward camera → Cruise control
Video stream (2)	Medium (SR class A)	1500	125	Rear camera → Center and HUD display
Video stream (3)	Medium (SR class A)	1250	125	Mirrorless camera (2) → Center and HUD display
V2X data	Medium (SR class A)	64	125	Integrated antenna → Cruise control
Entertainment data	Low (BE)	1500	1000	Integrated antenna → Seat monitor
Upload data	Low (BE)	100	1000	Tire pressure monitoring (2) → Integrated antenna

**Table 2:** Parameters for calculating the total length and weight of the wiring harnesses in the DIA and ZIAs.

Parameter	Values
Number of zones ($N_{Z})$	2, 4, 6, 8, 10
Number of domains ($N_{D})$	5
Number of ECUs ($N_{E})$	20, 40, 60, 80, 100, 120
Wiring harness weight per meter	0.0375 kg

## Data Availability

The original contributions presented in the study are included in the article, further inquiries can be directed to the corresponding author/s.

## References

[1] Leigh B., Duwe R. (2021). Designing autonomous vehicles for a future of unknowns. ATZelectronics Worldw..

[2] Delphi: Industry Must Go Digital or Die. https://www.wardsauto.com/technology/delphi-industry-must-go-digital-or-die.

[3] Van Rensburg P.J., Ferreira H.C. Automotive power-line communications: Favourable topology for future automotive electronic trends. Proceedings of the 7th International Symposium on Power-Line Communications and Its Applications (ISPLC’03).

[4] Rathore R.S., Hewage C., Kaiwartya O., Lloret J. (2022). In-vehicle communication cyber security: Challenges and solutions. Sensors.

[5] Askaripoor H., Hashemi Farzaneh M., Knoll A. (2022). E/E architecture synthesis: Challenges and technologies. Electronics.

[6] Brunner S., Roder J., Kucera M., Waas T. Automotive E/E-architecture enhancements by usage of ethernet TSN. Proceedings of the 2017 13th Workshop on Intelligent Solutions in Embedded Systems (WISES).

[7] Wang W., Yu S., Cao W., Guo K. (2022). Review of in-vehicle optical fiber communication technology. Automot. Innov..

[8] Oh S.B., Do Y.S., Jeon J.W. The Time Synchronization of CAN-FD and Ethernet for Zonal E/E Architecture. Proceedings of the 2023 International Technical Conference on Circuits/Systems, Computers, and Communications (ITC-CSCC).

[9] Park C., Park S. (2023). Performance evaluation of zone-based in-vehicle network architecture for autonomous vehicles. Sensors.

[10] Haeberle M., Heimgaertner F., Loehr H., Nayak N., Grewe D., Schildt S., Menth M. Softwarization of automotive E/E architectures: A software-defined networking approach. Proceedings of the 2020 IEEE Vehicular Networking Conference (VNC).

[11] Frigerio A., Vermeulen B., Goossens K.G. (2021). Automotive architecture topologies: Analysis for safety-critical autonomous vehicle applications. IEEE Access.

[12] Lo Bello L., Patti G., Leonardi L. (2023). A perspective on ethernet in automotive communications—Current status and future trends. Applied Sciences.

[13] Pannell D., Chen L., Dorr J., Lo W., Potts M., Zinner H., Zu A. (2022). Use Cases-IEEE P802.1DG V0. 4. https://www.ieee802.org/1/files/public/docs2019/dg-pannell-automotive-use-cases-0919-v04.pdf.

[14] Bandur V., Selim G., Pantelic V., Lawford M. (2021). Making the case for centralized automotive E/E architectures. IEEE Trans. Veh. Technol..

[15] (2020). Zonal Architecture: The Foundation for Next Generation Vehicles.

[16] IEEE802. https://www.ieee802.org/.

[17] Guo H. (2009). Automotive Informatics and Communicative Systems: Principles in Vehicular Networks and Data Exchange: Principles in Vehicular Networks and Data Exchange.

[18] Avatefipour O. (2017). Physical-Fingerprinting of Electronic Control Unit (ECU) Based on Machine Learning Algorithm for In-Vehicle Network Communication Protocol “CAN-BUS”. Master’s Thesis.

[19] Ignatious H.A., Khan M. (2022). An overview of sensors in Autonomous Vehicles. Procedia Comput. Sci..

[20] OMNeT++. https://omnetpp.org/.

[21] Core4INET—Real-Time Ethernet Protocols for INET. https://omnetpp.org/download-items/Core4INET.html.

[22] Lee J., Park S. (2019). Time-sensitive network (TSN) experiment in sensor-based integrated environment for autonomous driving. Sensors.

[23] Zhou Z., Lee J., Berger M.S., Park S., Yan Y. (2021). Simulating TSN traffic scheduling and shaping for future automotive Ethernet. J. Commun. Netw..

[24] Gopal A. Traffic categories & overall performance goals. In Proceedings of IEEE P802.1DG Interim Meeting. February 2022. https://www.ieee802.org/1/files/public/docs2022/dg-gopal-TrafficClassification-0222-v01.pdf.

[25] Thangamuthu S., Concer N., Cuijpers P.J., Lukkien J.J. Analysis of ethernet-switch traffic shapers for in-vehicle networking applications. Proceedings of the 2015 Design, Automation & Test in Europe Conference & Exhibition (DATE).

[26] Thangamuthu S. (2014). Analysis of Automotive Traffic Shapers in Ethernet In-Vehicular Networks. Master’s Thesis.

[27] Kurose J.F., Ross K.W., Anand B. (2008). Computer Networking: A Top-Down Approach.

[28] Stillig J., Parspour N. (2021). Novel infrastructure platform for a flexible and convertible manufacturing. Adv. Sci. Technol. Eng. Syst. J..

